# Efficacy of interventions to prevent eating disorders in people with type 1 diabetes: a systematic review

**DOI:** 10.1186/s40337-025-01460-2

**Published:** 2026-01-03

**Authors:** Neisha D’Silva, Nikka S. Sandoval, Kerri M. Gillespie, Line Wisting, Christel Hendrieckx, Eric Stice, Lee Jones, Sean N Gannon, Warren Ward, Melanie White, Selena E. Bartlett

**Affiliations:** 1 School of Clinical Sciences, Faculty of Health, Queensland University of Technology, Kelvin Grove, 4059 Brisbane, QLD Australia; 2 Mater Hospital Brisbane, Raymond Terrace, South Brisbane, 4101 South Brisbane, QLD Australia; 3 Mater Research, 4101 South Brisbane, QLD Australia; 4 Mater Research Institute-University of Queensland, 4067 St Lucia, QLD Australia; 5 The University of Queensland Medical School - Ochsner Clinical School, 4067 St. Lucia, QLD Australia; 6 Regional Department for Eating Disorders, Division of Mental Health and Addiction, Oslo University Hospital, 0450 Oslo, Norway; 7 Department of Psychology, Oslo New University College, 0456 Oslo, Norway; 8 School of Psychology, Deakin University, 3220 Geelong, VIC Australia; 9 The Australian Centre for Behavioral Research in Diabetes, Diabetes Victoria, 3053 Carlton, VIC Australia; 10 Department of Psychiatry and Behavioral Science, Stanford University, 94305 Palo Alto, USA; 11 QIMR Berghofer Medical Research Institute, 4006 Herston, QLD Australia; 12 The University of Queensland, 4006 Herston, QLD Australia; 13 School of Psychology and Counselling, Faculty of Health, Queensland University of Technology, 4059 Kelvin Grove, QLD Australia

**Keywords:** Type 1 diabetes, Eating disorder, Disordered eating, Intervention, Systematic review

## Abstract

**Background:**

Individuals with type 1 diabetes (T1D) are at increased risk of developing disordered eating (DE) and eating disorders (ED). Diabetes self-management focuses on food and insulin administration, which may contribute to development of EDs. The dual diagnosis may contribute to suboptimal glycemia, early diabetes-related complications and mortality. Evidence for ED prevention programs for this high-risk population remains limited. This systematic review aims to evaluate the feasibility and efficacy of available interventions to prevent EDs and improve glycemia in people with T1D.

**Methods:**

A literature search of PubMed, Embase, PsycINFO, CINAHL and Web of Science was conducted on 25 January 2025. Studies using randomized controlled, quasi-experimental or cohort design that targeted ED prevention in T1D population were included.

**Results:**

Nine studies met the inclusion criteria, featuring interventions such as cognitive dissonance based programs, psychoeducation and self-compassion programs. Cognitive dissonance based programs demonstrated the most consistent reduction in ED risks and symptoms. However, most studies showed negligible improvement in glycemia. Common limitations were small sample sizes, high drop-out rates and short follow-ups.

**Conclusion:**

Future research should focus on well-powered RCTs to evaluate interventions over longer timeframes, younger age groups, both genders, carer involvement and additional modifications to improve glycemia concurrently.

## Abstract (plain language summary)

People with type 1 diabetes (T1D) must pay continual attention to food consumption and insulin administration daily to self-manage their blood sugar levels. This constant focus on food can increase the risk of developing disordered eating behaviors/thoughts and eating disorders. When individuals are diagnosed with both T1D and an eating disorder, blood sugar levels are unstable, resulting in a much higher risk of early diabetes complications, hospitalization and shortened lifespan.

We reviewed studies that tested programs to prevent eating disorders in individuals with T1D. We found nine studies that included different types of programs, such as group discussions that challenge negative body image (cognitive dissonance based programs), psychology-based educational sessions, resilience and self-compassion workshops. Programs using cognitive dissonance approaches showed the most benefit in lowering eating disorder risk. However, none of the studies showed significant improvement in blood sugar levels.

Overall, research in this area is still limited. Many studies were small and short-term. More long-term, larger studies are needed to understand what works best, especially for younger individuals and across genders. Involving families and adjusting the programs to also support blood sugar management could help future programs be more effective.

## Introduction

The last 40 years have seen a greater awareness of the high prevalence of disordered eating (DE) and eating disorders (ED) in people with type 1 diabetes (T1D). The diagnostic criteria based on the DSM-5-TR [1], have been revised to include insulin omission, a unique ‘purging’ behavior employed by those with diabetes for weight loss (partially through inducing a catabolic state) [2]. DE occurs on a spectrum of severity from mildly disturbed thoughts and behaviors regarding weight, shape and eating to more concerning thoughts and behaviors with medical and mental health consequences that do not meet the formal criteria for an ED [3].

With the median age of onset at 14–19 years [4], the prevalence of DE in adolescent females with T1D may be as high as 40–50% and 13–18% in males [4, 5]. Adolescents with T1D are more than twice as likely to develop EDs compared to their peers without diabetes [4]. A recent systematic review and meta-analysis confirmed that T1D is associated with increased risk of ED compared with individuals without diabetes (RR = 2.47, 95% CI = 1.84–3.32, *p* < 0.00001) and reports a prevalence of insulin omission/restriction at 10.3%, predominantly in females [6]. This is partially due to the behavioral changes required for diabetes self-management (e.g. adjusting insulin doses with food intake and exercise) [7]. A longitudinal study found that 92% of female adolescents who reported DE at baseline continued to report DE at the 5-year follow-up, suggesting its persistent nature [8].

Prospective studies have identified risk factors that predict future onset of EDs, such as pressure for thinness, pursuit of the thin appearance ideal and body dissatisfaction [7, 9, 10]. Adolescents who report body dissatisfaction were more likely to restrict insulin, suggesting that this may be a particularly important risk factor for people with T1D [10]. Whilst females often strive for thinness, males may desire increased muscularity [5, 11]. In general, young people with T1D who perceive more significant disturbances with body image, shape and weight are more likely to omit insulin compared to those who do not, leading to glucose levels above target.

Evidence suggests that in addition to ED-specific complications, the dual diagnosis of T1D and ED (referred to as ED-T1D) can exponentially increase the risk of early onset of diabetes-related complications such as nephropathy, neuropathy and retinopathy [12]. Adolescents and young adults with T1D and ED have more than triple the risk of diabetic ketoacidosis (DKA) and nearly sixfold increased risk of death compared with their peers without eating disorders [13].

Local and international guidelines recommend regular assessment of psychological well-being in those with T1D [3, 14–17] with a view to early detection and management of DE to avoid the progression to an ED. The clinical inertia amongst health care professionals to screen, is partly due to a perceived lack of support and evidence-based interventions to prevent or treat DE in T1D [18].

A systematic review in 2017 [19] highlighted the limited evidence of interventions to prevent and/or treat DE or ED in T1D. After assessing the identified studies, based on the quality of intervention description and methodology, only six studies were deemed suitable for further analysis. Interventions identified were inpatient therapy, psychoeducation, cognitive behavioral therapy, multi-disciplinary approaches or family involvement. Whilst there were some improvements in ED psychopathology with some interventions, limited or no improvement in glycemia was observed.

Since this publication there have been substantial developments in interventions aimed at preventing ED-T1D. These have largely used cognitive dissonance theory to develop novel, co-designed programs that can be delivered in a variety of ways (such as online or with peer-facilitation). The methodologies in these studies vary from feasibility to large multicenter randomized controlled trials (RCT). Technological advancements in glucose monitoring (e.g., continuous glucose monitoring that measures glucose time-in-range) have allowed for better evaluation of interventions on glycemia. This systematic review evaluated the feasibility and efficacy of interventions to prevent ED and improve glycemia in people with T1D. Given the severe short- and long-term consequences of ED-T1D, there is a critical need for prevention strategies that target modifiable ED risk factors.

## Methods

The review follows Preferred Reporting Items for Systematic reviews and Meta-analyses (PRISMA 2020) and was registered with PROSPERO international prospective register of systematic reviews (ID: CRD420250649449).

### Search strategy

A comprehensive literature search (25 January 2025) was conducted across PubMed, Embase, PsycINFO, CINAHL and Web of Science (Appendix 1). Keywords included: “disordered eating”, “eating disorders”, “type 1 diabetes”, “intervention”. Subject headings (e.g. MeSH in PubMed) were employed. The search was limited to English-language, peer-reviewed publications. Citation chaining and hand searches identified no additional studies. Previous and current systematic reviews were searched in the databases listed, Cochrane Library and PROSPERO registry. One prior systematic review (2017) addressed ED prevention and treatment for people with T1D [19]. Our systematic review updates and narrows the focus to prevention, including two studies from that review.

### Inclusion and exclusion criteria

- *Included*: Randomized Control Trials (RCT), quasi-experimental and cohort studies reporting primary data on ED prevention in T1D.
- *Excluded*: Cross-sectional, case-control, qualitative studies; non-primary data (reviews, commentaries, abstracts); non-English papers; studies aimed at treating (versus preventing) participants with either established ED or ED determined by a comprehensive clinical assessment using DSM-5-TR.

### Study selection

Two authors (ND and NS) independently screened titles/abstracts in Covidence. Disagreements were resolved by a third reviewer (KG). Full-text articles were reviewed similarly.

### Data extraction

Two authors (ND and NS) extracted data independently, with biostatistician input for accuracy. Extracted data included author, year, country, design, sample details, intervention/ control conditions, clinical measures, outcomes and study strengths/limitations.

#### Clinical measures

- *Person-reported outcome measures PROMs*: diabetes-specific ED symptoms, body dissatisfaction, thin-ideal internalization, diabetes distress and general psychopathology (symptoms of anxiety/ depression, general well-being).
- *Glycemia measures*: HbA1c (mean glucose concentration over 8–12 weeks) and TIR (percentage of time in target glucose range, >70% considered optimal [20]). Studies under 3 months’ duration were unable to assess HbA1c meaningfully.

### Data synthesis and quality assessment

Meta-analysis was not feasible due to heterogeneity in design, intervention, population (particularly different age ranges and gender inclusion) and outcomes, making pooling results and direct comparisons difficult. Further, the number of identified studies was too small to provide sufficient statistical power for a meta-analytic review. A narrative synthesis was conducted. Studies were evaluated using the Joanna Briggs Institute (JBI) tools: 13-item (RCTs), 9-item (quasi-experimental), and 11-item (cohort). Two reviewers assessed quality; discrepancies were resolved by a third. Although quality was not used as an exclusion criterion due to the limited number of identified studies, JBI scores (refer to Appendix 2) were used to inform the interpretation and weighting of findings in the narrative synthesis.

## Results

### Study selection

**Fig. 1:**
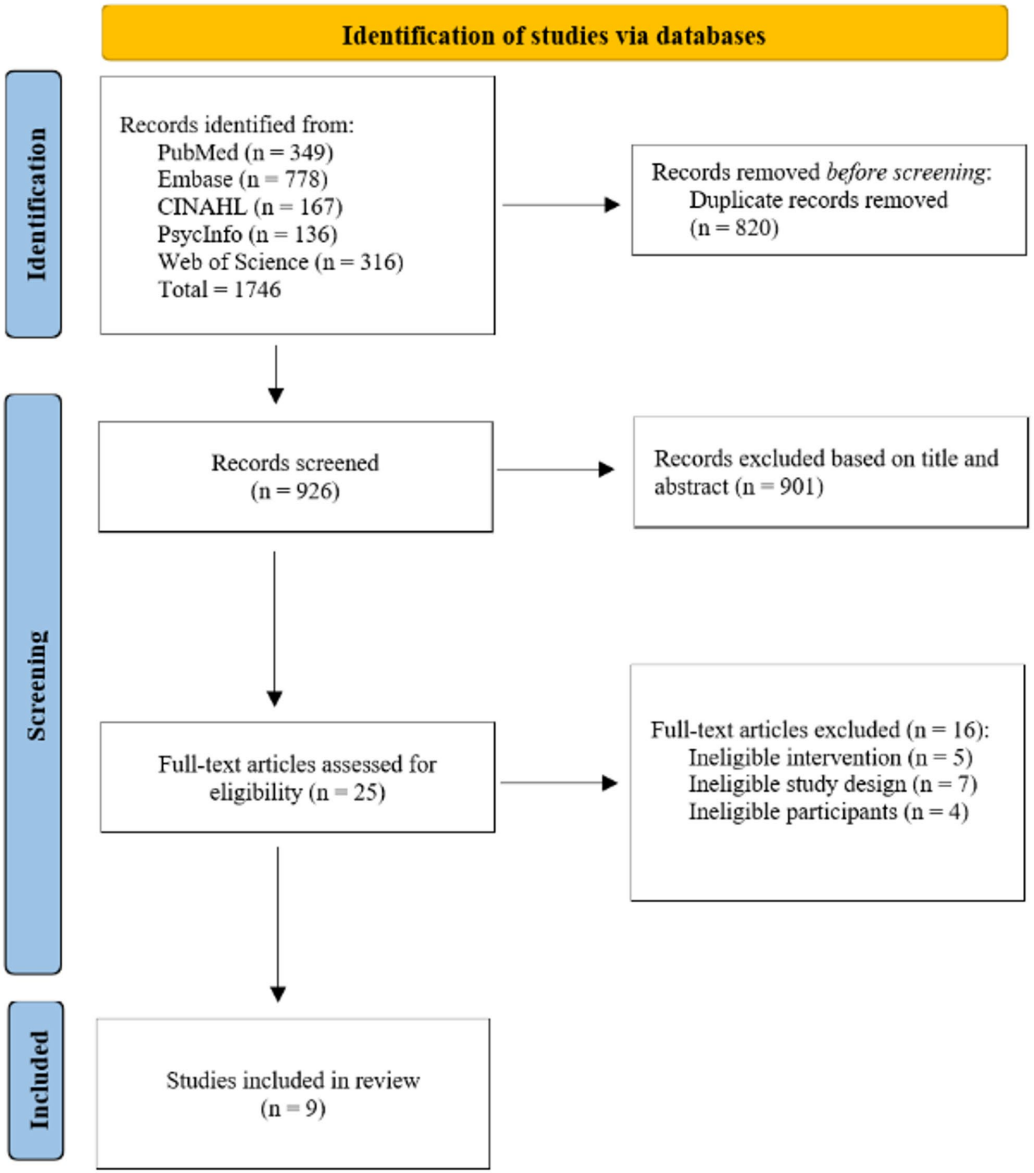
PRISMA flow diagram

From 1,746 records, 926 remained after de-duplication. Title/abstract screening yielded 25 full-texts; 9 studies met inclusion criteria [21–29]. No additional studies were identified through citation chaining and hand-searching. (Fig. 1)

### Quality assessment

Of the nine studies, methodological quality varied considerably. While one quasi-experimental study [28] achieved the maximum JBI score, indicating strong internal validity, others—particularly feasibility and pilot RCTs—scored lower due to limitations such as lack of blinding, small sample sizes, and short follow-up durations. Studies employing cognitive dissonance based interventions generally demonstrated higher methodological rigor, supporting the robustness of their findings on ED symptoms. The absence of blinding, particularly in psychoeducation and self-compassion interventions, raised concerns about bias. Full detail has been tabulated in Appendix 2.

### Characteristics of included studies

#### Study design

Among the nine studies (Table 1): five were RCTs [22–26], two quasi-experimental [21, 28] and two were cohort studies [27, 29]. Three RCTs were feasibility trials. Studies were geographically diverse, with two conducted in Oceania [22, 28], four in North America [21, 25–27], two in Europe [24, 29], and one as a multinational collaboration between Europe and North America [23].

Recruitment strategies included social media, third-sector organizations (e.g. diabetes associations) and diabetes clinics.

#### Sample characteristics

Most studies recruited females with only one study including both sexes [22]. Age ranges varied considerably (reported individually in Table 1) with only four studies aimed at those less than 14 years [22, 24, 25, 28]. Sample sizes ranged from 14 to 293 with five studies having under 50 participants. Four studies incorporated family or carer involvement [24, 25, 27, 28] though conclusions are limited due to small sample size and age variability. Five out of the nine studies reported on ethnicity. Participants were predominantly ‘white,’ ranging between 59% [27] to 90% [24], and averaging 75% across the studies. No comparison of study outcome measures was reported based on ethnicity.

#### Interventions

Four studies used psychoeducation (resilience, body acceptance, media literacy, perfectionism, or self-esteem) [21, 24, 25, 28]; one used a self-compassion program [22] and four used cognitive dissonance based programs [23, 26, 27, 29]. The latter intervention is designed to create cognitive dissonance to reduce a key attitudinal risk factor (pursuit of the thin-ideal), which increases the risk for an ED. The cognitive dissonance based program was adapted for people with T1D from the Body Project program, an efficacious ED prevention program for the general population [30, 31]. Three of these studies used the *Diabetes Body Project* for ages 14–35 [23, 26, 29] and one used the *Body Project T1D* for adolescents aged 15–18 with caregiver sessions [27]. Five interventions were virtual [23, 24, 26, 27, 29], four in-person [21, 22, 25, 28]; none were delivered individually. Delivery mode effectiveness was inconclusive due to heterogeneity. Controls included usual care [22, 24, 25] or active educational video content [23, 26].

Drop-out rates measured from consent/randomization to follow-up, ranged from 0 to 40%. Higher retention was seen in studies with younger participants, parental involvement, and shorter durations, although this was inconsistent across studies. No pattern was found by intervention type.

Finally, three studies incorporated peer facilitators with T1D [23, 26, 29]. All three reported statistically significant improvements in ED-related symptoms and risk. However, as each also employed cognitive dissonance based methods, it is difficult to disentangle the independent effects of peer delivery from the intervention itself.

### Clinical measures

#### Diabetes-specific ED symptoms

Diabetes-specific ED symptoms were primarily assessed using the Diabetes Eating Problem Survey-Revised (DEPS-R), a validated 16-item questionnaire used widely in clinical and research settings [32, 33]. It assesses general and diabetes-specific disordered eating behaviors including weight loss, dietary restriction, insulin omission, and vomiting.

Six studies included in this review utilised the DEPS-R. Four of the six studies, employed cognitive dissonance based interventions, and reported improvements; two showed significantly lower mean DEPS-R scores in intervention groups compared to controls: *d* = -0.7 [23] and *d* = -0.74 [26], another reported a significant reduction within-group at 6-month follow-up (*d* = -0.7) [29], and the fourth using the *Body Project T1D* showed a within-group improvement at 3-month follow-up (*d* = -0.49) [27]. The remaining studies did not report any statistical inferences [22, 24].

#### Body dissatisfaction

Body dissatisfaction is a well-established risk factor for EDs and was assessed with the Eating Disorder Inventory (EDI) with its body dissatisfaction subscale, the Body Dissatisfaction Scale (BD), the Satisfaction and Dissatisfaction with Body Parts Scale (BPS) [34], and the Screen for Early Eating Disorder Signs (SEEDS). Five studies employed one or more of these instruments [23, 25–27, 29]. All reported improvements in body dissatisfaction at follow-up. Four of these studies used cognitive dissonance based interventions [23, 26, 27, 29], and one used psychoeducation [25]. Improvements were consistent across all studies measuring this outcome, with effect sizes ranging from moderate *d* = -0.59 [27] to large *d* = -0.76 [29] for within-group effects, and from *d* = -0.46 [23] to *d* = -0.59 [26] for between-group differences.

#### Diabetes distress

Diabetes distress was assessed in six studies using validated tools, including Problem Areas in Diabetes (PAID: age-appropriate versions) [22, 23, 27, 29], , PAID-PR (parent-version) [24] and Diabetes Distress Scale (DDS) [26]. Cognitive dissonance based programs showed small to moderate improvements between-groups (e.g., *Diabetes Body Project: d*=-0.42 post-intervention [23]; *d*=-0.17 at 3-month follow-up [26]). Within-group improvements were also observed at 3-months (*d*=-0.49) [27] and 6-months (*d*=-0.84) [29]. No change was noted in parent-reported diabetes distress following a psychoeducation program (*d*=-0.07) [24] and one study found a non-significant increase in distress following a self-compassion program (mean change = + 2.0, *SD* = 7.9) [22].

#### Depressive symptoms

Depressive symptoms were assessed in three studies using interventions of psychoeducation programs [21, 28] and the *Diabetes Body Project* [26]. While some improvements were observed in depressive symptoms, these were generally modest and not statistically significant.

#### Glycemic measures

Glycemic outcomes included HbA1c and time-in-range (TIR). In three studies, follow-up durations were less than three months [21, 22, 28], making HbA1c outcomes unreliable due to the timeframe required to observe clinically meaningful changes. Among the remaining studies that reported post-intervention glycemic outcomes, none demonstrated significant improvements in HbA1c [25, 27] or TIR [23, 26].

**Table 1:** Characteristics of the studies

Author/ year/ country	Study design and aim	Recruitment	Sample characteristics	Intervention	Follow-up period	Clinical measures	Major findings	Study strengths and study limitations
Alloway et al., 2001 [21] Canada	Quasi-experimental Aim: Evaluate efficacy of 6-week psychoeducation program on the primary outcomes of metabolic control, ED symptomatology, general psychopathology and diabetes treatment adherence.	Community and diabetes clinic.	Female age 20+ Subclinical DE (based on EDI and EAT scores) Exclusion: self-reported bingeing with any compensatory behavior twice a week for 3-months, or BMI < 20. *N* = 14 I: *n* = 8, age 32.5 (9.3) C: *n* = 6, age 31 (10.3)	Psychoeducation: -Education on ED -Normal eating and healthy body weight -Assertiveness, stress management and self-esteem -Media influence, perfectionism and body image 6x weekly Group sessions In-person Facilitator: dietitian Waitlist control	1-month (both groups) 6-month (treatment group only)	BDI BSI EAT EDI Insulin omission RSES HbA1c (%) fructosamine	No statistically significant effect of intervention on any outcomes. Between-group effects at 1-month follow-up: BDI: MD = -15.7 BSI: MD = -0.8 EAT: MD = -13.2 EDI: MD = -1.1 Insulin omission: MD = -1 RSES: MD = -5.8 HbA1c: MD = -0.1%	Limitations: Small sample size Self-selection bias Older participants (average age 31.9) Short follow-up Participants non-blinded. Control group had significantly greater general emotional distress (BSI) at baseline (*p* = 0.009) Strengths: 14/14 completed (0% drop-out at 1-month).
Boggiss et al., 2020 [22] New Zealand	RCT (feasibility), although results are reported as single-group pre-post study Aim: Assess feasibility and acceptability of a self-compassion program	Diabetes clinic	Male or female age 12–16 Mod to high DEB on DEPS-R (no inclusion/ exclusion scores provided) N – 27 were randomized *N* = 19 completed study I: *n* = 11, age 14 (1.2), F = 7 C: *n* = 8, age 13.6 (1.3), F = 3	Self-compassion program *“Making friends with yourself”* 2x weekly Group sessions (2.5 h per session), In-person Facilitator: clinical psychologist Waitlist control	2-week	Primary outcome: Feasibility/ acceptability qualitative measures Secondary outcome: DEPS-R PAID PSS SCS-SF SCI-R HbA1c (%)	Pre-post measures from both groups pooled and reported as within-group effects Within-group effects immediately post-intervention: DEPS-R: Mean change = + 0.3 (5.8 SD) PAID: Mean change = + 2.0 (7.9 SD) PSS Mean change = + 0.9 (6.1 SD) SCS-SF Mean change = + 0.1 (0.5 SD) SCI-R Mean change = + 1.5 (8.4 SD) HbA1c: Mean change = -0.2%	Limitations: Small sample size Low attendance: ~ 50% attended both sessions. Short follow-up, particularly to note any metabolic improvement No between-group comparisons reported Strengths: 19/27 completed (30% drop-out)
Hennekes et al., 2025 [23] US and Europe	RCT Aim: Evaluate efficacy of Diabetes Body Project (DBP) on primary outcomes of ED symptoms and behaviors, psychological constructs and glycemia outcomes	Social media and Diabetes clinic	Female age 14–35 Report body image concerns. Exclusion: ED-related hospitalization or DKA *N* = 293 I: *n* = 147, age 25.2 (5.43) C: *n* = 146, age 24.8 (5.58)	Cognitive dissonance based program *Diabetes Body Project* 6 x weekly Group sessions (1-hour per session) Online Facilitator: diabetes clinicians and peer educator. Active control (Educational videos)	1–2 week	BD DEPS-R DEBQ-RS EDDI TIIS PAID PANAS T1DAL TIR	Between-group effects immediately post-intervention (adjusted for pre-intervention scores and study site): BD: aMD = -0.32 (*d* = -0.59, *p* < 0.001) DEPS-R: aMD = -4.04 (*d* = -0.7, *p* < 0.001) DEBQ-RS: aMD = -0.18 (*d* = -0.32, *p* = 0.021) EDDI: aMD = -1.95 (*d =* -0.3, *p* = 0.026) TIIS: aMD = -0.31 (*d* = -0.31, *p* < 0.001) PAID: aMD = -4.71 (*d* = -0.42, *p* = 0.002) PANAS: back-transformed mean difference not reported (*d* = -0.23, *p* = 0.091) T1DAL: aMD = + 3.21 (*d* = 0.39, *p* = 0.004) TIR: back-transformed mean difference not reported (*d* = -0.08, *p* = 0.544)	Limitations: Short duration follow-up Selection bias for digitally literate (social media) Strengths: Large sample size 265/293 (9.6% drop out).
Jones et al., 2024 [24] UK	RCT (feasibility - parent program) Aim: Evaluate feasibility, acceptability and preliminary efficacy of a psychoeducational program for parents of children with T1D on DEPS-R as the primary outcome.	Diabetes care teams and 3rd sector organizations	Parents with children age 11–14 *N* = 89 parents I: *n* = 44 (child’s age 8.52 +/- 3.12) C: *n* = 45 (child’s age 7.89 +/- 3.39)	Psychoeducation: *PRIORITY* intervention for parents -communication around food and weight -recognizing early DE -supporting identity and independence in T1D 2x weekly Group sessions (2-hour sessions) Online Facilitator: psychologist Waitlist control	3-month	Primary outcome: Parent and child reported DEPS-R Secondary outcome: CEBQ: 8 subscales PAID-PR WEMWBS Only baseline HbA1c	Between-group effects at 3-month follow-up: Parent-reported DEPS-R: *d* = -0.1 (95% CI -0.63 to 0.43) Child-reported DEPS-R: *d* = -0.5 (95% CI -1.26 to 0.3) CEBQ: No significant effect for any of the 8 subscales except Satiety Responsiveness, *d* = + 0.55 (95% CI 0.01 to 1.08) PAID-PR: *d* = -0.07 (95% CI -0.6 to 0.46) WEMWBS: *d* = + 0.27 (95% CI -0.27 to 0.79)	Limitations: Short follow-up 55/89 completed (38% dropout rate) Effect sizes not reported on original scale Strengths: Moderate sample size Robust methodology with independent randomization, blinded outcome assessment
Olmstead et al., 2002 [25] Canada	RCT Aim: Evaluate the effectiveness of a psychoeducational program on ED psychopathology, ED risk factors, insulin omission and glycemia	Diabetes clinic	Females age 12–20 Inclusion: DE based on EDI or DSED scores. *N* = 85 age 16 (2.0) I: *n* = 50 C: *n* = 35	Psychoeducation: Didactic information on healthy eating, DE and T1D. Parent and daughters 6x weekly Group sessions (90-minute sessions) In-person Facilitators: ED clinician and adolescent DM clinician Control: Usual care	6-month	EDE, EDI, Insulin omission HbA1c (%)	Between-group effects over 6 months of follow-up: EDE: Restraint: *p* = 0.004 Overeating: NS Eating concern: *p* = 0.05 Shape concern: NS Weight concern: NS EDI: Drive for thinness: *p* = 0.03 Bulimia: NS Body dissatisfaction: *p* = 0.008 Insulin omission days: NS HbA1c: NS	Limitations: 6% (*n* = 3) drop-out in psychoeducation arm, 31.4% (*n* = 11) in usual care Strength: Moderate sample size
Stice et al., 2023 [26] USA	RCT (pilot) Aim: Evaluate whether Diabetes Body Project can reduce ED risk factor and symptoms, diabetes distress and improve quality of life.	Social media/email	Females age 15–30 Report body image concerns. Exclusion: ED-related hospitalization or DKA *N* = 55 I: *n* = 30; age 23.5 (3.83) C: *n* = 25; age 22.06 (4.66)	Cognitive dissonance based program *Diabetes Body Project* 6x weekly Group sessions (1-hour per session) Online Facilitators: diabetes clinician and peer educator. Active control (Educational video)	3-month	BD, DDS DEPS-R, EDDI, TII, WHO-5 TIR (where available)	Between-group effects at 3-month follow-up (adjusted for pre-intervention scores): BD: aMD = -0.24, *d* = -0.35, *p* = 0.121 DDS: aMD = -0.18, *d* = -0.17, *p* = 0.336 DEPS-R: aMD = -5.6, *d* = -0.58, *p* = 0.002 EDDI: aMD = -0.72, *d* = -0.46, *p* = 0.013 TII: aMD = -0.21, *d* = -0.31, *p* = 0.116 WHO-5: aMD = + 0.46, *d* = 0.46, *p* = 0.038 TIR: aMD = + 0.05, *d* = 0.26, *p* = 0.126	Limitations: Limited power of pilot study Short follow-up. No HbA1c measure Strength: Moderate sample size.
Trojanowski et al., 2022 [27] USA	Longitudinal cohort (feasibility) Aim: Evaluate feasibility, acceptability and preliminary efficacy of Body Project T1D on ED risk factor and symptoms, diabetes distress and quality of life	Diabetes clinic	Females age 15–18 *N* = 35, age 16.2 (1.12) Exclusion: active ED diagnosis noted by medical staff.	Cognitive dissonance based program *Body Project T1D.* Caregiver session 4x weekly Group sessions (60–90 min sessions) Online Facilitators: 2x psychologists	3-month	DEPS-R, SEEDS, DREBQ, SATAQ-4, PAID-T, DAS, PedsQL	Within-group effects at 3-month follow-up: DEPS-R: MD = -4.06, *d* = -0.49 (-0.81, -0.19) SEEDS: MD = -7.43, *d* = -0.58 (-0.85, -0.32) DREBQ: MD = -0.49, *d* = -0.60 (-0.95, -0.25) PAID-T: MD = -28.58, *d* = -0.49 (-1.50, -0.80) SATAQ-4 (thin/low body fat subscale) = -0.65, *d* = -0.58 (-0.23, -0.85) DAS: MD = -5.24, *d* = -0.73 (-1.06, -0.36) PedsQL: MD = -4.66, *d* = -0.42 (-0.74, -0.04)	Limitations: Cohort study. No controls Small sample size Strength: Minimal drop-out rate
Wilksch et al., 2013 [28] Australia	Quasi- experimental Aim: Evaluate the effectiveness of a psychoeducation program that enhances protective factors for ED.	Diabetes clinic.	Females age 10–12 *N* = 20, age 11.06 (0.64)	Psychoeducation: program targeting perfectionism, media literacy and self-esteem 2x weekly Group sessions (4-hour session), Parents invited to separate sessions Facilitator: psychologist and diabetes nurse educator 4-week control period, followed by intervention	1-month	Primary outcome: CDI-SF, BES-C, DRFQ MPS, SATAQ-3, SEDS, RSES Secondary outcome: HbA1c (%)	Within-group effects at 1 month follow-up (adjusted for pre-intervention scores): CDI-SF: Not reported BES-C: aMD = + 0.13, *d* = 0.32 DRFQ: Not reported MPS: aMD = -0.35, *d* = -0.65 SATAQ-3: aMD = -0.46, *d* = -0.4 SEDS: aMD = + 0.34, *d* = 0.56 RSES: aMD = + 0.31, *d* = 0.57 HbA1c: aMD = + 0.11%, *d* = + 0.13, not significant.	Limitations: Small sample size Short follow-up period Strengths: 0% dropout
Wisting et al., 2024 [29] Norway	Longitudinal cohort (pilot) Aim: Evaluate within -subject changes in ED risk factors and symptoms in those who received the Diabetes Body Project program.	Social media, diabetes organization	Female age 16–35 Report body image concerns. Exclusion: ED-related hospitalization or DKA *N* = 24; age 25.62 (3.95)	Cognitive dissonance based program *Diabetes Body Project* 6x weekly Group sessions (1-hour per session) Online Facilitator: diabetes clinicians and peer educator.	6-month	Primary outcomes: IBSS-R, BPS, DEPS-R, PANAS-X, DRES. Secondary outcomes: BIPQ PAID, SATAQ-4R, SCS HbA1c (%): self-reported	Within-group effects at 6-month follow-up: IBSS-R: Mean change = -0.23 (95% CI -0.43 to -0.03), *d* = -0.43 BPS: Mean change = -0.45 (95% CI -0.66 to -0.25), *d* = -0.76 DEPS-R: Mean change = -5.24 (95% CI -7.6 to -2.9), *d* = -0.70 PANAS-X: Mean change = -5.3 (95% CI -10.5 to -0.07), *d* = -0.38 DRES: Mean change = -0.46 (95% CI -0.71 to -0.2), *d* = -0.55 BIPQ: Mean change = -3.7 (95% CI -6.05 to -1.35), *d* = -0.56 PAID: Mean change = -10.1 (95% CI -14.9 to -5.3), *d* = -0.84 SATAQ-4R: Mean change = -11.2 (95% CI -16.9 to -5.4), *d* = -0.60 SCS: Mean change = + 3.2 (95% CI -1.3 to + 7.7), *d* = + 0.27 HbA1c (%): Mean change = -0.57% (95% CI -0.31% to + 0.20%), *d* = -0.13. The Cohen’s *d* values presented here have been recalculated from original data using Eq. 2.3.5 of Cohen [35], to facilitate comparison with other studies.	Limitations: Cohort study Small sample size Dropout rate 31.4%

*aMD*: Adjusted Mean Difference, *BD*: Body Dissatisfaction, *BDI*: Beck Depression Inventory, *BES-C*: Body-Esteem Scale for Children, *BIPQ*: Brief Illness Perception Questionnaire, *BPS*: Body Parts Scale, *BSI*: Brief Symptom Inventory, C: Control group, *CDI-SF*: Children’s Depression Inventory - Short Form, *CEBQ*: Child Eating Behavior Questionnaire, d: Cohen’s *d*, *DAS*: Diabetes Acceptance Scale, *DDS*: Diabetes Distress Scale, *DEBQ-RS*: Dutch Eating Behavior Questionnaire - Restrained Eating Scale, *DEPS-R*: Diabetes Eating Problem Survey – Revised, *DREBQ*: Dutch Restrained Eating Behaviors Questionnaire, *DRES*: Diabetes-Related Eating Symptoms, *DRFQ*: Diabetes Family Responsibility Questionnaire, *DSED*: Diagnostic Survey for Eating Disorders, *EAT*: Eating Attitudes Test, *EDDI*: Eating Disorder Diagnostic Interview, *EDE*: Eating Disorder Examination, *EDE-QS*: Eating Disorder Examination Questionnaire - Short, *EDI*: Eating Disorder Inventory, F: Female, *GAD-7*: Generalized Anxiety Disorder-7, I: Intervention group, *IBSS-R*: Ideal-Body Stereotype Scale - Revised, *MD*: Mean Difference, *MPS*: Multidimensional Perfectionism Scale, *PAID*: Problem Areas in Diabetes, *PAID-PR*: Problem Areas in Diabetes - Parent Revised, *PAID-T*: Problem Areas in Diabetes - Teen, *PANAS*: Positive and Negative Affect Schedule, *PANAS-X*: Positive and Negative Affect Schedule - Revised, Parental Self-Efficacy for Diabetes, *PedsQL*: Pediatric Quality of Life - Core Scales, *PHQ-9*: Patient Health Questionnaire-9, *PSS*: Perceived Stress Scale, *RSES*: Rosenberg Self-Esteem Scale, *SATAQ-3*: Sociocultural Attitudes Toward Appearance Questionnaire, 3rd edition, *SATAQ-4*: Sociocultural Attitudes Toward Appearance Questionnaire, 4th edition, *SATAQ-4R*: Sociocultural Attitudes Toward Appearance Questionnaire, 4th edition Revised, *SCI-R*: Self-Care Inventory - Revised, *SCS*: Social Comparison Scale, *SCS-SF*: Self-Compassion Scale - Short Form, *SEDS*: Self-Efficacy for Diabetes Scale, *SEEDS*: Screen for Early Eating Disorder Signs, *T1DAL*: Type 1 Diabetes and Life, *TIIS / TII*: Thin-Ideal Internalization Scale, *WEMWBS*: Warwick Edinburgh Mental Wellbeing Scale, *WHO-5*: World Health Organization Well-Being Index

## Discussion

This systematic review evaluated the feasibility and efficacy of interventions aimed at preventing ED in individuals with T1D and improving glycemia. The heterogeneity in methodological quality, as assessed using JBI tools, underscores the need for cautious interpretation of findings.

Most studies employed DEPS-R to assess change in diabetes-specific ED symptomatology. Cognitive dissonance based interventions reported statistically and clinically significantly lower mean DEPS-R scores in the intervention group, both between-groups and within-groups. These results underscore the potential of cognitive dissonance based techniques to reduce maladaptive eating behaviors and cognitions in T1D, putatively by reducing valuation of the thin-ideal. Due to overlapping variables—such as peer-facilitation in *Diabetes Body Project* or caregiver involvement in *Body Project T1D* which could potentially influence outcomes—causality remains unclear. Nonetheless, these results are promising.

Five studies assessed body dissatisfaction using the Eating Disorder Inventory (EDI), the Body Dissatisfaction Scale (BD), the Satisfaction and Dissatisfaction with Body Parts Scale (BPS) or the Screen for Early Eating Disorder Signs (SEEDS). Improvements were consistent across all studies measuring this outcome, with moderate to large effect sizes for within-group effects, and small to medium effect sizes for between-group effects when compared to a control group. Four of these five studies also utilised cognitive dissonance based approaches, reinforcing their value in addressing and enhancing body acceptance.

Cognitive dissonance based interventions also demonstrated moderate to large reductions in diabetes distress, with effects strengthening over time. In contrast, psychoeducation and self-compassion programs showed no improvements, though these studies were likely limited by study design and sample size. Symptoms of depression outcomes showed modest, non-significant improvements across interventions, suggesting that while some psychological benefits may occur, targeted strategies and longer follow-up are needed to meaningfully impact depressive symptoms in this population.

Metabolic outcomes such as HbA1c and time-in-range (TIR) were largely unaffected by the interventions. In four studies, the follow-up duration was less than three months, rendering HbA1c changes physiologically implausible and uninterpretable. In the remaining studies with follow-ups up to 6-months, no intervention demonstrated statistically significant improvements in glycemia. This suggests that while psychological interventions may successfully address cognitive and behavioral precursors to EDs, they do not directly improve metabolic outcomes in the short term. This is unexpected as there is a correlation between ED-T1D and insulin omission, suboptimal glycemic outcome, recurrent DKA and consequent diabetes-related complications [12, 35]. Insulin administration is the most potent influence on glycemia management. Whilst the DEPS-R incorporates a question on insulin omission, scoring of the tool does not specifically focus on this compensatory behavior. Future studies should analyse insulin omission separately, to gain further insight as to whether interventions are targeting this aspect of ED “purging” behavior which directly influences glycemic outcomes.

The integration of peer facilitators with lived experience of T1D was another notable feature of successful programs. Peer-led interventions appeared more relatable and may have enhanced outcomes, particularly in the adolescent and young adult populations. However, the overlap between peer delivery and cognitive dissonance content makes it difficult to disentangle the relative contributions of each component to the observed effects. Nonetheless, this combination appears to offer a promising model for future interventions.

Furthermore, family involvement—present in four studies—was associated with improved retention, particularly in younger populations. For instance, a paediatric intervention with concurrent parent sessions reported 0% drop-out [28]. However, inconsistency in participant age ranges, intervention formats and outcome reporting make it difficult to isolate and assess the specific impact of caregiver involvement on ED risk reduction in these four studies. Without consistent measurement of family dynamics or stratification by developmental stage, the role of caregiver participation in modifying ED risk remains unclear.

Overall, the cognitive dissonance based interventions appear promising in preventing ED in those with T1D. However, given that the onset of ED symptoms occur from age 14 (some would argue even younger), this program requires adaptation and evaluation in the paediatric space.

This systematic review applied rigorous standards in accordance with PRISMA 2020 and was prospectively registered on PROSPERO, which enhanced transparency and methodological accountability. A broad and inclusive search strategy, with the initial inclusion of both DE and established ED, across multiple databases, with citation chaining and hand searches for peer-reviewed papers, maximized the likelihood of capturing relevant studies. A major methodological strength was the dual-review process with conflict resolution and data extraction conducted independently by multiple reviewers, with the involvement of a biostatistician to ensure analytical clarity. This reduced risk of bias in study selection and data synthesis.

However, several limitations should be noted. First, the decision not to conduct a meta-analysis—whilst justified due to heterogeneity, as well as the relatively small number of studies —limits the ability to quantify overall intervention effects. Second, the exclusion of non-English studies may have geographically concentrated the locations to North America, Europe, and Oceania, with no representation from Asia, Africa, or South America. This exclusion and the fact that study participants were predominantly of European descent, limits the generalizability of findings to non-Western populations, especially considering cultural differences in body ideals.

The predominance of female participants is reflective of ED epidemiology but leaves male populations underrepresented, limiting gender-based conclusions. Recruitment through social media and healthcare settings may also bias the sample toward individuals with better access to resources and higher health literacy.

Most of the studies had small samples and high dropout rates (20–40%). This weakens statistical power as well as raises concerns about selection bias. These challenges highlight important considerations for future research. Interventions may benefit from being brief, co-designed with participants to improve acceptability, incorporating flexible delivery formats (e.g., digital or hybrid models), and including motivational or family-based components to support ongoing engagement. Embedding retention strategies, such as reminders, incentives, or tailored content may also help reduce dropout and improve study feasibility.

Finally, while all included studies utilised validated clinical tools, the diversity in outcome selection, duration of follow-up, and absence of standardised behavioral metrics complicates cross-study comparisons. Most follow-up periods were under six months, restricting conclusions about the long-term sustainability of intervention effects.

## Conclusion

In summary, this review suggests that structured psychological interventions—particularly those using cognitive dissonance based strategies—may reduce key ED risk factors and ED pathology in people with T1D. Improvements were most notable in diabetes-specific ED symptomatology and body dissatisfaction, while glycemic outcomes remained unchanged. Though limited by sample size, heterogeneity, and geographic scope, this review provides valuable insight into the potential of preventive strategies. Future research should focus on larger, more diverse samples, younger age groups, longer follow-up durations, and integration of both psychological and metabolic outcomes to better understand the sustained impact of these interventions across global T1D populations.

## Data Availability

No datasets were generated or analysed during the current study.

## Appendix 1

**Full search strategy**.

**PubMed 25th January 2025 (349 articles)**.

[Title/abstract] (Intervention* OR program OR programme OR psychotherap* OR psychoeducation OR therapy OR counselling OR treatment* OR inpatient*) AND (“disordered eating” OR “eating disorder*” OR bulimia OR anorexia OR “insulin omission” OR diabulimia OR “eating problem” OR “eating psychopathology” OR bing* OR purg* OR restrict*) AND (“type 1 diabet*” OR “diabetes mellitus type 1” OR T1D OR T1DM OR IDDM OR “insulin dependent” OR ketoacidosis).

MeSH terms.

diabetes mellitus, type 1.

(feeding and eating disorders of childhood OR eating disorders)

English only.

**EMBASE 28th 25th January 2025 (778 articles)**.

Title and Abstract (Intervention* OR program OR programme OR psychotherap* OR psychoeducation OR therapy OR counselling OR treatment* OR inpatient*) AND (“disordered eating” OR “eating disorder*” OR bulimia OR anorexia OR “insulin omission” OR diabulimia OR “eating problem” OR “eating psychopathology” OR bing* OR purg* OR restrict*) AND (“type 1 diabet*” OR “diabetes mellitus type 1” OR T1D OR T1DM OR IDDM OR “insulin dependent” OR ketoacidosis).

Subject headings.

‘eating disorder’/exp.

‘insulin dependent diabetes mellitus’/exp.

English only.

**CinAHL 25th January 2025 (167 articles)**.

Title and Abstract (Intervention* OR program OR programme OR psychotherap* OR psychoeducation OR therapy OR counselling OR treatment* OR inpatient*) AND (“disordered eating” OR “eating disorder*” OR bulimia OR anorexia OR “insulin omission” OR diabulimia OR “eating problem” OR “eating psychopathology” OR bing* OR purg* OR restrict*) AND (“type 1 diabet*” OR “diabetes mellitus type 1” OR T1D OR T1DM OR IDDM OR “insulin dependent” OR ketoacidosis).

Subject Headings.

DE “Eating Disorders” OR DE “Anorexia Nervosa” OR DE “Avoidant/Restrictive Food Intake Disorder” OR DE “Binge Eating Disorder” OR DE “Bulimia” OR DE “Orthorexia” OR DE “Purging.

MM “Type 1 Diabetes”.

English only.

**PsycInfo 25th January 2025 (136 articles)**.

Title (Intervention* OR program OR programme OR psychotherap* OR psychoeducation OR therapy OR counselling OR treatment* OR inpatient*) AND (“disordered eating” OR “eating disorder*” OR bulimia OR anorexia OR “insulin omission” OR diabulimia OR “eating problem” OR “eating psychopathology” OR bing* OR purg* OR restrict*) AND (“type 1 diabet*” OR “diabetes mellitus type 1” OR T1D OR T1DM OR IDDM OR “insulin dependent” OR ketoacidosis).

Subject headings.

(MA “Eating Disorders+”)

(MA “Diabetes Mellitus, Type 1+”)

English only.

**Web of Science 25th January 2025 (316 articles)**.

All Fields (Intervention* OR program OR programme OR psychotherap* OR psychoeducation OR therapy OR counselling OR treatment* OR inpatient*) AND (“disordered eating” OR “eating disorder*” OR bulimia OR anorexia OR “insulin omission” OR diabulimia OR “eating problem” OR “eating psychopathology” OR bing* OR purg* OR restrict*) AND (“type 1 diabet*” OR “diabetes mellitus type 1” OR T1D OR T1DM OR IDDM OR “insulin dependent” OR ketoacidosis).

English only.

**Final search – 1**,**746**.

**De-duplication − 926**.

## Appendix 2

**Quality assessment of included Studies**.

JBI - Joanna Briggs Institute.

**Table:**

Author, Year	Study Type	Q1	Q2	Q3	Q4	Q5	Q6	Q7	Q8	Q9	Q10	Q11	Q12	Q13	Total Point
Alloway, 2001	Quasi- experimental	1	1	0	1	1	1	1	0	0.5					6.5/9
Boggis, 2020	RCT feasibility	1	1	0	0	0	0	1	1	0	1	0	0	0	5 /13
Hennekes, 2025	RCT	1	0	0	1	0	1	1	1	1	1	1	1	1	10/13
Jones, 2004	RCT feasibility	1	0	0	0	0	1	1	1	1	0	1	0	0	6/13
Olmstead, 2002	RCT	1	1	1	0	0	1	0.5	1	1	1	1	1	1	10.5/13
Stice, 2023	RCT pilot	1	1	1	0	1	1	1	1	1	1	1	1	1	11/13
Trojanowski, 2022	Longitudinal (feasibility) cohort	1	0	0	0	0	1	1	1	1	1	1			6/11
Wilksch, 2013	Quasi- experimental	1	1	1	1	1	1	1	1	1					9/9
Wisting, 2024	Longitudinal (pilot) cohort	1	0	1	1	0	0	1	1	1	0	1			7/11

JBI Critical Appraisal Checklist for Randomized Controlled Trials (RCTs):

1. Was true randomization used for assignment of participants to treatment groups?
2. Was allocation to treatment groups concealed?
3. Were treatment groups similar at baseline?
4. Were participants blind to treatment assignments?
5. Were those delivering treatment blind to treatment assignment?
6. Were outcome assessors blind to treatment assignment?
7. Were treatment groups treated identically other than the intervention?
8. Was follow-up complete, and if not, were differences accounted for?
9. Were participants analyzed in the groups to which they were randomized (intention-to-treat)?
10. Were outcomes measured in the same way for treatment groups?
11. Were outcomes measured reliably?
12. Was appropriate statistical analysis used?
13. Was the trial design appropriate, and were any deviations from the protocol reported?

JBI Critical Appraisal Checklist for Quasi-Experimental Studies.

1. Is it clear what the “cause” and what is the “effect” (i.e. there is no confusion about which variable comes first)?
2. Was there a control group?
3. Were participants included in any comparisons similar?
4. Were the participants included in any comparisons receiving similar treatment/care, other than the exposure or intervention of interest?
5. Were there multiple measurements of the outcome, both pre and post the intervention/exposure?
6. Were the outcomes of participants included in any comparisons measured in the same way?
7. Were outcomes measured in a reliable way?
8. Was follow-up complete and if not, were differences between groups in terms of their follow-up adequately described and analyzed?
9. Was appropriate statistical analysis used?

JBI Critical Appraisal Checklist for Cohort Studies.

1. Were the two groups similar and recruited from the same population?
2. Were the exposures measured similarly to assign people to both exposed and unexposed groups?
3. Was the exposure measured in a valid and reliable way?
4. Were confounding factors identified?
5. Were strategies to deal with confounding factors stated?
6. Were the groups/participants free of the outcome at the start of the study?
7. Were the outcomes measured in a valid and reliable way?
8. Was the follow-up time reported and sufficient for outcomes to occur?
9. Was follow-up complete, and if not, were losses to follow-up described and explored?
10. Were strategies to address incomplete follow-up utilized?
11. Was appropriate statistical analysis used?

## References

[1] American Psychiatric Association. Diagnostic and statistical manual of mental disorders. 5th ed. Text Revision. Washington, DC: American Psychiatric Publishing; 2022.

[2] Vidyasagar S, Griffin A, d’Emden H, Hendrieckx C, D’Silva N. Perceived comfort with weight, body shape and eating pattern of young adults with type 1 diabetes and associations with clinical and psychological parameters in a clinical setting. J Eat Disord. 2024;12(1):106. 10.1186/s40337-024-01059-z. 39080802 10.1186/s40337-024-01059-z PMC11289989

[3] Disordered eating. and Eating disorders in children, adolescents and adults with type 1 diabetes. Qld Diabetes Clin Netw; 2022; 12: 106.

[4] Young V, Eiser C, Johnson B, et al. Eating problems in adolescents with type 1 diabetes: a systematic review with meta-analysis. Diabet Med. 2013;30(2):189–98. 10.1111/j.1464-5491.2012.03771.x. 22913589 10.1111/j.1464-5491.2012.03771.x

[5] Araia E, Hendrieckx C, Skinner T, Pouwer F, Speight J, King RM. Gender differences in disordered eating behaviors and body dissatisfaction among adolescents with type 1 diabetes: results from diabetes MILES youth-Australia. Int J Eat Disord. 2017;50(10):1183–93. 10.1002/eat.22746. 28856699 10.1002/eat.22746

[6] Dean YE, Motawea KR, Aslam M, et al. Association between type 1 diabetes mellitus and eating disorders: a systematic review and meta-analysis. Endocrinol Diabetes Metab. 2024;7(3):e473. 10.1002/edm2.473. 38597269 10.1002/edm2.473 PMC11005101

[7] Toni G, Berioli MG, Cerquiglini L, et al. Eating disorders and disordered eating symptoms in adolescents with type 1 diabetes. Nutrients. 2017;9(8):908. 10.3390/nu9080906. 28825608 10.3390/nu9080906 PMC5579699

[8] Colton PA, Olmsted MP, Daneman D, Farquhar JC, Wong H, Muskat S, et al. Eating disorders in girls and women with type 1 diabetes: a longitudinal study of prevalence, onset, remission, and recurrence. Diabetes Care. 2015;38(7):1212–7. 10.2337/dc14-2646. 25887359 10.2337/dc14-2646

[9] Rohde P, Stice E, Marti CN. Development and predictive effects of eating disorder risk factors during adolescence: implications for prevention efforts. Int J Eat Disord. 2015;48(2):187–98. 10.1002/eat.22270. 24599841 10.1002/eat.22270 PMC4156929

[10] Ackard DM, Vik N, Neumark-Sztainer D, Schmitz KH, Hannan P, Jacobs DR Jr. Disordered eating and body dissatisfaction in adolescents with type 1 diabetes and a population-based comparison sample: comparative prevalence and clinical implications. Pediatr Diabetes. 2008;9(4 Pt 1):312–9. 10.1111/j.1399-5448.2008.00392.x. 18466215 10.1111/j.1399-5448.2008.00392.x

[11] Ricciardelli LA, McCabe MP. A biopsychosocial model of disordered eating and the pursuit of muscularity in adolescent boys. Psychol Bull. 2004;130(2):179–205. 10.1037/0033-2909.130.2.179. 14979769 10.1037/0033-2909.130.2.179

[12] Peveler RC, Bryden KS, Neil HA, Fairburn CG, Mayou RA, Dunger DB, et al. The relationship of disordered eating habits and attitudes to clinical outcomes in young adult females with type 1 diabetes. Diabetes Care. 2005;28(1):84–8. 10.2337/diacare.28.1.84. 15616238 10.2337/diacare.28.1.84

[13] Gibbings NK, Kurdyak PA, Colton PA, Shah BR. Diabetic ketoacidosis and mortality in people with type 1 diabetes and eating disorders. Diabetes Care. 2021;44(8):1783–7. 10.2337/dc21-0517. 34172488 10.2337/dc21-0517

[14] Craig ME, Twigg SM, Donaghue KC, et al. National evidence-based clinical care guidelines for type 1 diabetes in children, adolescents and adults. Canberra: Australian Government Department of Health and Ageing; 2011.

[15] National Institute for Health and Care Excellence. Type 1 diabetes in adults: diagnosis and management (NICE Guideline NG17). 2022. Available from: https://www.nice.org.uk/guidance/ng17 32017485

[16] de Wit M, Gajewska KA, Goethals ER, et al. ISPAD clinical practice consensus guidelines 2022: psychological care of children, adolescents and young adults with diabetes. Pediatr Diabetes. 2022;23(11):1373–89. 10.1111/pedi.13428. 36464988 10.1111/pedi.13428 PMC10107478

[17] Young-Hyman D, de Groot M, Hill-Briggs F, Gonzalez JS, Hood K, Peyrot M. Psychosocial care for people with diabetes: a position statement of the American diabetes association. Diabetes Care. 2016;39(12):2126–40. 10.2337/dc16-2053. 27879358 10.2337/dc16-2053 PMC5127231

[18] Fitzgerald K, Jones C, Partridge H, Rouse L, Satherley RM. Exploring healthcare professionals’ attitudes to screening for disordered eating in type 1 diabetes. Diabet Med. 2025;e70003. 10.1111/dme.70003. 10.1111/dme.70003 PMC12006562 39948754

[19] Clery P, Stahl D, Ismail K, Treasure J, Kan C. Systematic review and meta-analysis of the efficacy of interventions for people with type 1 diabetes mellitus and disordered eating. Diabet Med. 2017;34(12):1667–75. 10.1111/dme.13509. 28887815 10.1111/dme.13509

[20] Battelino T, Danne T, Bergenstal RM, et al. Clinical targets for continuous glucose monitoring data interpretation: recommendations from the international consensus on time in range. Diabetes Care. 2019;42(8):1593–603. 10.2337/dci19-0028. 31177185 10.2337/dci19-0028 PMC6973648

[21] Alloway SC, Toth EL, McCargar LJ. Effectiveness of a group psychoeducation program for the treatment of subclinical disordered eating in women with type 1 diabetes. Can J Diet Pract Res. 2001;62(4):188–92. 11742560

[22] Boggiss AL, Consedine NS, Schache KR, et al. A brief self-compassion intervention for adolescents with type 1 diabetes and disordered eating: a feasibility study. Diabet Med. 2020;37(11):1854–60. 10.1111/dme.14352. 32614482 10.1111/dme.14352

[23] Hennekes MHCL, Haugvik S, de Wit M, et al. Diabetes body project: acute effects of an eating disorder prevention program for young women with type 1 diabetes. A multinational randomized controlled trial. Diabetes Care. 2024;48(2):220–5. 10.2337/dc24-1599. 10.2337/dc24-1599 39602474

[24] Jones CJ, Read R, O’Donnell N, et al. PRIORITY trial: results from a feasibility randomized controlled trial of a psychoeducational intervention for parents to prevent disordered eating in children and young people with type 1 diabetes. Diabet Med. 2024;41(4):e15263. 10.1111/dme.15263. 38100228 10.1111/dme.15263

[25] Olmsted MP, Daneman D, Rydall AC, Lawson ML, Rodin G. The effects of psychoeducation on disturbed eating attitudes and behavior in young women with type 1 diabetes mellitus. Int J Eat Disord. 2002;32(2):230–9. 10.1002/eat.10068. 12210667 10.1002/eat.10068

[26] Stice E, Wisting L, Desjardins CD, et al. Evaluation of a novel eating disorder prevention program for young women with type 1 diabetes: a preliminary randomized trial. Diabetes Res Clin Pract. 2023;206:110997. 10.1016/j.diabres.2023.110997. 37951479 10.1016/j.diabres.2023.110997 PMC11326084

[27] Trojanowski PJ, Frietchen RE, Harvie B, Mehlenbeck R, Fischer S. Internet-delivered eating disorders prevention program for adolescent girls with type 1 diabetes: acceptable and feasible. Pediatr Diabetes. 2022;23(7):1122–32. 10.1111/pedi.13395. 35869788 10.1111/pedi.13395 PMC9804811

[28] Wilksch SM, Starkey K, Gannoni A, Kelly T, Wade TD. Interactive programme to enhance protective factors for eating disorders in girls with type 1 diabetes. Early Interv Psychiatry. 2013;7(3):315–21. 10.1111/eip.12012. 23347796 10.1111/eip.12012

[29] Wisting L, Haugvik S, Wennersberg AL, et al. A pilot study of a virtually delivered dissonance-based eating disorder prevention program for young women with type 1 diabetes: within-subject changes over 6-month follow-up. Eat Disord. 2024;32(6):686–702. 10.1080/10640266.2024.2331391. 38511886 10.1080/10640266.2024.2331391

[30] Stice E, Marti CN, Shaw H, Rohde P. Meta-analytic review of dissonance-based eating disorder prevention programs: intervention, participant, and facilitator features that predict larger effects. Clin Psychol Rev. 2019;70:91–107. 10.1016/j.cpr.2019.04.004. 31004832 10.1016/j.cpr.2019.04.004 PMC6536334

[31] Stice E, Rohde P, Shaw H, Gau JM. Clinician-led, peer-led, and internet-delivered dissonance-based eating disorder prevention programs: effectiveness of these delivery modalities through 4-year follow-up. J Consult Clin Psychol. 2020;88(5):481–94. 10.1037/ccp0000493. 32091226 10.1037/ccp0000493 PMC7810349

[32] Markowitz JT, Butler DA, Volkening LK, Antisdel JE, Anderson BJ, Laffel LM. Brief screening tool for disordered eating in diabetes: internal consistency and external validity in a contemporary sample of pediatric patients with type 1 diabetes. Diabetes Care. 2010;33(3):495–500. 10.2337/dc09-1890. 20032278 10.2337/dc09-1890 PMC2827495

[33] Pursey KM, Hart M, Jenkins L, McEvoy M, Smart CE. Screening and identification of disordered eating in people with type 1 diabetes: a systematic review. J Diabetes Complications. 2020;34(4):107522. 10.1016/j.jdiacomp.2020.107522. 31928891 10.1016/j.jdiacomp.2020.107522

[34] Berscheid E, Walster E, Bohrnstedt G. The happy American body: a survey report. Psychol Today. 1973;7(6):119–31.

[35] Nielsen S, Emborg C, Molbak AG. Mortality in concurrent type 1 diabetes and anorexia nervosa. Diabetes Care. 2002;25(2):309–12. 10.2337/diacare.25.2.309. 11815501 10.2337/diacare.25.2.309

